# Face it or hide it: parental socialization of reappraisal and response suppression

**DOI:** 10.3389/fpsyg.2013.00992

**Published:** 2014-01-03

**Authors:** Catherine Gunzenhauser, Anika Fäsche, Wolfgang Friedlmeier, Antje von Suchodoletz

**Affiliations:** ^1^Department of Psychology, Research Group “The Empirics of Education”, University of FreiburgFreiburg, Germany; ^2^Psychology Department, Grand Valley State UniversityAllendale, MI, USA

**Keywords:** emotion socialization, parenting, cognitive emotion regulation strategies, reappraisal, response suppression

## Abstract

Mastery of cognitive emotion regulation strategies is an important developmental task. This paper focuses on two strategies that occur from preschool age onwards (Stegge and Meerum Terwogt, 2007): reappraisal and response suppression. Parental socialization of these strategies was investigated in a sample of *N* = 219 parents and their children. Informed by the tripartite model of family impact on children's emotion regulation, direct relations of emotion socialization components (modeling and reactions to the child's negative emotions) and indirect relations of parental emotion-related beliefs (such as parental emotion regulation self-efficacy) were examined. Data on emotion socialization components and parental beliefs on emotion regulation were collected via self-report. Data on children's emotion regulation strategies were collected via parent report. Findings showed direct effects of parental modeling and parenting practices on children's emotion regulation strategies, with distinct socialization paths for reappraisal and response suppression. An indirect effect of parental emotion regulation self-efficacy on children's reappraisal was found. These associations were not moderated by parent sex. Findings highlight the importance of both socialization components and parental emotion-related beliefs for the socialization of cognitive emotion regulation strategies and suggest a domain-specific approach to the socialization of emotion regulation strategies.

## Introduction

The children's soccer team loses the last game of the season after a tough match. Six-year-old players Michael and Jacob are fighting to hold back their tears when leaving the pitch to meet their parents. Michael's father puts his arm around his son's shoulders and tries to cheer him up by praising Michael's and his teammates' great shots. After a while, Michael says that he thinks his team played well although they lost. Jacob's mother seems very composed. She tells her son that a lost game is not a big deal and that Jacob is behaving like a baby. Her son tries hard not to cry.

In the above example, Michael *reappraised* the situation and thus changed its emotional meaning. Jacob *suppressed* the external signs of his disappointment. Individual differences in the use of reappraisal and response suppression have far-reaching consequences in emotional, social, and cognitive domains; for instance, they are associated with mental and physical health, and with general life satisfaction in adults (John and Gross, 2004). Therefore, it is important to understand socialization influences that may shape individual differences in the development of habitual use of these cognitive emotion regulation strategies. John and Gross (2004) suggested that parental emotion-related beliefs and behaviors might play a crucial role in the development of habitual reappraisal and response suppression use in children. The tripartite model of family impact on children's emotion regulation (Morris et al., 2007) offers a theoretical framework to specify and examine this assumption. Informed by the tripartite model, the present study investigated direct and indirect relations between parental emotion socialization and child reappraisal and response suppression as reported by parents. Moreover, moderating effects of parent sex were explored.

Emotion regulation refers to “attempts individuals make to influence which emotions they have, when they have them, and how these emotions are experienced and expressed” (Gross et al., 2006, p. 14). The diverse ways in which individuals undertake emotion regulation can be referred to as emotion regulation strategies (Koole, 2009). In most instances, emotion regulation aims at decreasing the experience or expression of a negative emotional state (Gross et al., 2006). The current study focused on two widely used cognitive emotion regulation strategies: *reappraisal* and *response suppression* (Gross and John, 2003). Reappraisal refers to a cognitive reframing of a situation, usually taking a neutral perspective or trying to adopt a more positive view of things. Response suppression describes the effortful inhibition of external signs of an ongoing emotion. Both reappraisal and response suppression can be effective in adjusting an emotional experience or expression to situational demands (Thompson and Meyer, 2007). However, there are individual differences in adults' habitual use of reappraisal and response suppression that are stable across emotions with positive and negative valence (Gross and John, 2003).

Experimental and longitudinal studies with adolescents and adults have revealed that individuals from Western cultural backgrounds who are habitual reappraisers tend to be in better health than people who are not (John and Gross, 2004; Gullone and Taffe, 2012). Moreover, habitual reappraisers tend to experience positive emotions more frequently and build more satisfying personal relationships. In contrast, habitual response suppression is associated with an increased experience of negative emotions and depressiveness in both adolescents and adults (John and Gross, 2004; Gullone and Taffe, 2012). In addition, studies with adults have revealed an association between habitual response suppression use and undesirable cognitive consequences in terms of memory impairments (John and Gross, 2004). Thus, in Western cultures such as the U.S. or Western Europe, habitual reappraisal can be conceptualized as overall adaptive, and habitual response suppression can be viewed as overall maladaptive (see John and Gross, 2004; Butler et al., 2007; Abler and Kessler, 2009; McRae et al., 2012).

During their preschool and early elementary school years, children acquire cognitive skills that lay the foundation for the use of a wide array of cognitive emotion regulation strategies (Cole et al., 2009). Physiological and structural changes in the prefrontal cortex improve preschool children's ability to inhibit responses (Center on the Developing Child at Harvard University, 2011), thereby facilitating the response suppression of emotional expression. Simultaneously, preschoolers' developing theory of mind allows them to understand that a person's overt emotional expression does not necessarily reflect this person's true feelings (Wellman et al., 2001). At the same time, preschool children come to understand that changing thoughts can change emotions (Harris and Lipian, 1989). Consistently, reappraisal and response suppression occur from preschool age onwards, although developmental psychological literature often uses slightly different concepts and terms to describe related phenomena (Cole, 1986; Thompson, 1991; Stansbury and Sigman, 2000; Davis et al., 2010; for a review, see Stegge and Meerum Terwogt, 2007). From a theoretical perspective, John and Gross (2004) suggested that parental emotion-related beliefs and behaviors might play a pivotal role in the development of habitual reappraisal and response suppression use. However, to our knowledge, parental socialization of individual differences in reappraisal and response suppression has not yet been empirically investigated in early and middle childhood (for a review see Bariola et al., 2011).

**Figure 1:**
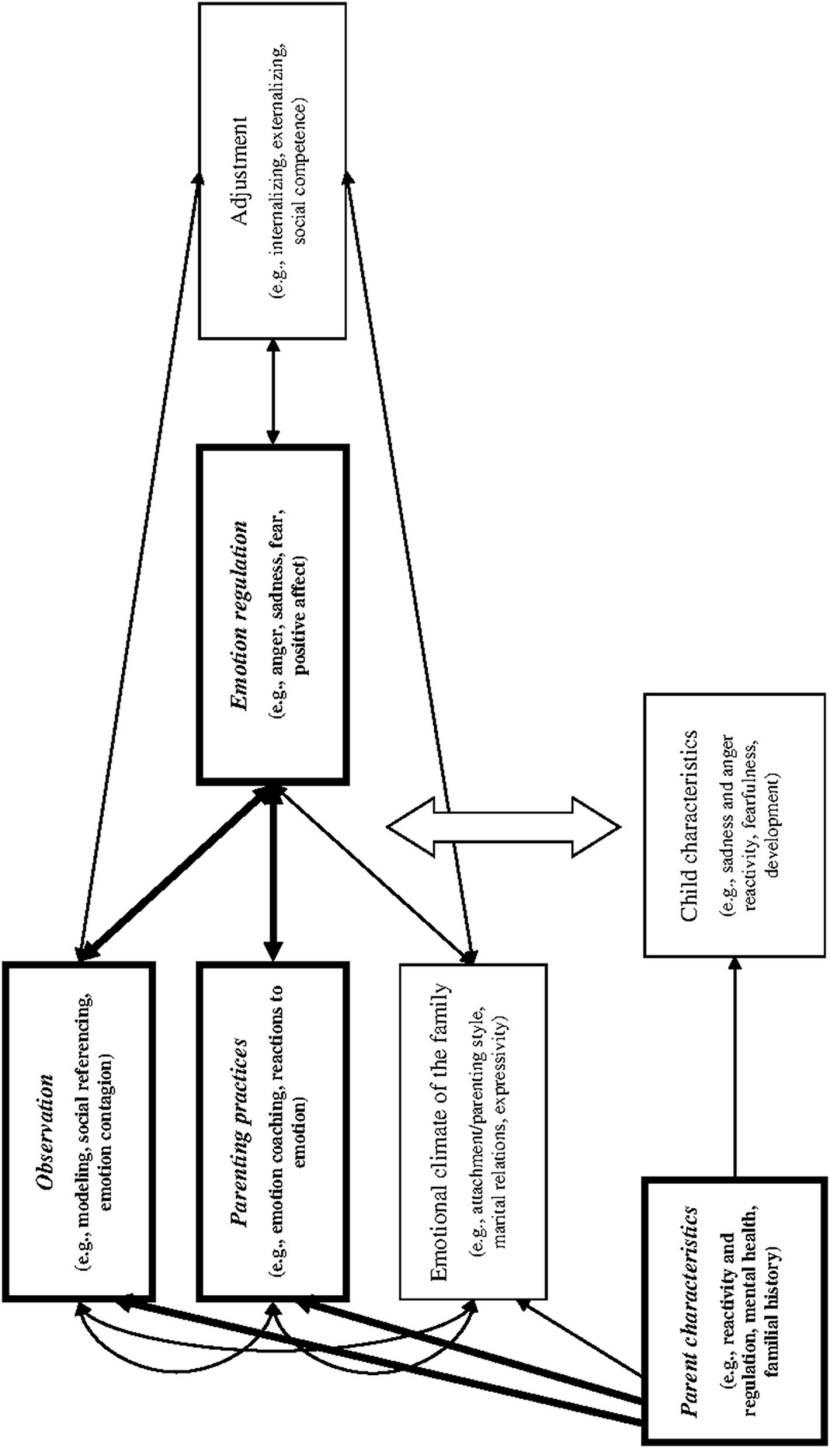
**The Tripartite model of family impact on children's emotion regulation and adjustment**. Figure adapted from Morris et al. (2007, p. 362). The parts of the model investigated in the present study are printed bold.

The tripartite model of familial impact on children's emotion regulation and adjustment (see Figure 1) summarizes in which ways children learn about emotion regulation in the family (Morris et al., 2007). Building on an integrative review of the current literature, Morris et al. (2007) suggested that children's emotion regulation is directly influenced by three socialization components: observation/modeling, emotion-related parenting practices, and emotional climate of the family. Moreover, the tripartite model assumes that parent characteristics (such as parents' own emotion regulation) exert indirect influences on children's emotion regulation through the three socialization components.

Notably, the tripartite model is not limited to describing parental emotion-related beliefs and behaviors, but includes parent characteristics beyond emotion-related beliefs (e.g., parents' mental health), takes into account the broader family context (as exemplified in the inclusion of family climate), and extends to children's socio-emotional adjustment over and above emotion regulation strategies (Morris et al., 2007). Nevertheless, the part of the tripartite model that focuses on parental characteristics, parental modeling, and parenting practices (see Figure 1) describes specific socialization influences of individual mothers' and fathers' emotion-related beliefs and behaviors on child emotion regulation. Thus, the tripartite model provides a framework to investigate parental socialization of reappraisal and response suppression as assumed by John and Gross (2004).

The first emotion socialization component described in the tripartite model is parental modeling (Thompson, 1991; Denham et al., 1997; see Bandura, 1977). Morris et al. (2007) suggested that parents' own use of emotion regulation strategies serves as a model for children. In empirical studies, parents' use of emotion regulation strategies that involve avoiding negative thoughts and emotions have been linked to ineffective emotion regulation strategies and internalizing behavioral symptoms in preschool-aged children (Silk et al., 2006; Coyne and Thompson, 2011). For instance, Silk et al. (2006) compared emotion regulation strategies used by children of depressed and non-depressed mothers in a delay task, considering depressed mothers as being impaired in their modeling of adaptive emotion regulation strategies as a concomitant of their affective disorder. In this study, daughters of depressed mothers as compared to daughters of non-depressed mothers were more likely to focus on the delay or wait passively, both of which were considered maladaptive strategies (Silk et al., 2006). Interestingly, daughters of depressed mothers were also less likely to engage in distraction, a strategy considered to be adaptive (Silk et al., 2006). Thus, parental use of particular emotion regulation strategies might not only facilitate children's use of this particular strategy but also hinder children's use of other strategies. To our knowledge, relations between parents' and their children's use of reappraisal and response suppression have so far not been investigated with children in early and middle childhood. However, in a study with nine- to 19-year-olds and their parents, Bariola et al. (2012) found a significant positive association between maternal use of response suppression and their children's use of response suppression.

The second emotion socialization component mentioned in the tripartite model are parenting practices. Although parental reactions to children's negative emotions are only one aspect of emotion-related parenting practices, they have been shown to be strongly related to various child emotion regulation outcomes (Eisenberg et al., 1998). Parents differ in their tendency to show supportive and non-supportive reactions in response to their children's expression of emotions (Gottman et al., 1996; Eisenberg et al., 1998; Davidov and Grusec, 2006; McElwain et al., 2007). Supportive and non-supportive reactions are usually negatively related to one another but not mutually exclusive (see Eisenberg et al., 1998).

Central features of *supportive reactions* include practices that communicate that the child's emotions are legitimate and may focus on the child's emotions or the problem itself (Eisenberg et al., 1998). Supportive parental reactions teach children how to constructively address distressing situations and contribute to child emotional competence (Gottman et al., 1996; Eisenberg et al., 1998; Fabes et al., 2002). At the same time, there is some evidence relating parental supportive reactions to expression of negative emotions in children (see Eisenberg et al., 1998), implying that supportive reactions might hinder children's response suppression. *Non-supportive reactions* include reactions that devaluate the child's emotions and do not provide any means of reducing emotional arousal but communicate that emotional displays are to be avoided (Eisenberg et al., 1998). Consistently, Fabes et al. (2001) reported evidence that parental non-supportive reactions are related to a lower frequency, but higher intensity of children's negative emotional expression. Also, parental non-supportive reactions have been linked to a lack of social skills and adaptive coping (Eisenberg et al., 1996).

The role of parental reactions to children's emotions for children's reappraisal and response suppression has rarely been empirically investigated. An exception is a study by Jaffe et al. (2010), who linked adolescents' reappraisal and response suppression use to their retrospective ratings of parental care, a unidimensional construct related to supportive and non-supportive reactions. Results revealed that higher perceived parental care was related to adolescents' reappraisal use, whereas lower perceived parental care predicted adolescents' response suppression.

Morris et al. (2007) suggested that parents' characteristics are likely to guide their modeling and parenting practices. Thereby, parental characteristics exert indirect influences on children's emotion regulation. John and Gross (2004) suggested that parental emotion-related beliefs might be crucial parental characteristics with regard to the socialization of reappraisal and response suppression. In line with this assumption, the present study focused on parents' emotion regulation self-efficacy concerning the management of negative emotions, that is, the perceived capability to change emotional states and to avoid being overcome by negative emotions such as anger or sadness (Caprara et al., 2008). Parents' emotion regulation self-efficacy is a parental emotion-related characteristic that targets specifically emotion regulation, and has been shown to shape behavior (Bandura, 1997; Caprara et al., 2008). Emotion regulation self-efficacy may relate to an increased likelihood of engaging in emotion regulation strategies that involve facing the emotional experience such as reappraisal (Gunzenhauser et al., 2013). Consistently, several studies have found positive associations between emotion regulation self-efficacy and reappraisal use in adults (Tamir et al., 2007; Carthy et al., 2010). In contrast, emotion regulation self-efficacy and response suppression have found to be unrelated in adults (Tamir et al., 2007). Emotion regulation self-efficacy can also affect emotion-related parenting practices (Caprara and Steca, 2005; Hoffmann, 2008). Adults with high emotion regulation self-efficacy are more likely to react in empathic ways to others' emotions (Caprara and Steca, 2005). Parents with high emotion-regulation self-efficacy can thus be expected to be able to react in more supportive ways when their child experiences negative emotions, and to be less likely to show non-supportive reactions. Consistently, parents' self-efficacy was shown to predict supportive reactions and a lack of non-supportive reactions in a study with German mothers and their 4–7-year old children (Hoffmann, 2008).

Differences between men and women have been reported for response suppression as well as for parental supportive and non-supportive reactions, while there is no evidence for sex differences in reappraisal and emotion regulation self-efficacy (Gross and John, 2003; Abler and Kessler, 2009; Wong et al., 2009). At the same time, parent sex differences in the associations between emotion socialization components and child emotion regulation have been suggested (Gottman et al., 1996; Wong et al., 2009). However, the nature and direction of these differences remain unclear. On the one hand, there is some evidence that mothers tend to be more involved in their children's emotion socialization, thus exerting greater influences on children's emotion regulation than fathers (McDowell et al., 2002; McElwain et al., 2007; Bariola et al., 2011). On the other hand, fathers' modeling and parenting practices may be more strongly related to their emotion-related beliefs such as perceived self-efficacy because paternal role expectations are less scripted (see Wong et al., 2009). Empirically, recent studies on parent sex differences in the relations between parenting and child outcomes have yielded mixed results, with sex differences confirmed for specific emotion-related socialization components and child outcomes but not for others (McElwain et al., 2007; Wong et al., 2009; Baker et al., 2011; Liang et al., 2012). For instance, McElwain et al. (2007) found no parent sex differences regarding relations between parental emotion socialization and children's understanding of mixed emotions, but they found fathers' emotion socialization to be more strongly related to children's emotion false-belief understanding than mothers'. In the study by Wong et al. (2009), mothers' and fathers' non-supportive behaviors and emotional expressiveness related differentially to their child's negative emotionality, but only when parental resources (more accepting beliefs on children's emotions and less marital conflict) were high. No parent sex differences were found between parent emotion-related beliefs and their emotion-related parenting behavior (Wong et al., 2009). Thus, the present research explored whether there are differences between mothers and fathers with regard to direct and indirect relations between parental emotion socialization components, parental emotion regulation self-efficacy, and child emotion regulation strategies.

Informed by the tripartite model (Morris et al., 2007), the present study examined parental socialization of two cognitive emotion regulation strategies: children's reappraisal and response suppression. While the few previous studies on the socialization of reappraisal and response suppression analyzed socialization components separately and focused on older children, the present study took several components as well as emotion regulation self-efficacy into account and analyzed their direct and indirect relations with emotion regulation strategies in early and middle childhood. Focusing on parental socialization (see John and Gross, 2004), the study addressed three aims: (1) to investigate direct associations of socialization ‘components (i.e., parental modeling and parental reactions to children's negative emotions) with child reappraisal and response suppression, (2) to examine indirect associations of mothers' and fathers' emotion-related beliefs (i.e., emotion regulation self-efficacy) with child reappraisal and response suppression, and (3) to explore moderating effects of parent sex on the relations between emotion socialization components, parental emotion regulation self-efficacy, and child emotion regulation strategies.

Specifically, we expected that parents' reappraisal modeling, their supportive reactions, and a lack of non-supportive reactions would be positively related to child reappraisal. In contrast, we suggested that parents' response suppression modeling, their non-supportive reactions and a lack of supportive reactions would be positively related to child response suppression. In addition, we explored possible negative relations between parents' response suppression modeling and child reappraisal (parents' reappraisal modeling on child response suppression, respectively). This was based on research findings that parental modeling of particular emotion regulation strategies might not only facilitate children's use of this particular strategy but also hinder children's use of other strategies (Silk et al., 2006).

We expected parents' emotion regulation self-efficacy to be indirectly related to children's emotion regulation strategies through parental modeling and parental reactions to children's negative emotions. It was anticipated that higher parental self-efficacy would relate to higher child reappraisal in part through parental reappraisal modeling, parental supportive reactions, and a lack of parental non-supportive reactions. In turn, higher parental self-efficacy would relate to lower levels of child response suppression in part through the same pattern of parental modeling and parental reactions. Based on previous research showing no relationship between emotion regulation self-efficacy and response suppression in adults (Tamir et al., 2007), no indirect relations of parental self-efficacy through parental response suppression modeling were expected. Nevertheless, it was explored whether such relations might exist. Finally, evidence on parent sex differences in regard to socialization of emotion regulation is both scarce and mixed (Gottman et al., 1996; McElwain et al., 2007; Wong et al., 2009). Therefore, parent sex was investigated as a possible moderator of all direct and indirect relations in the model.

## Materials and methods

### Participants

In the present study, *n* = 117 mothers and *n* = 102 fathers of *N* = 118 children (51% girls) participated. The sample included *N* = 101 parental couples. All families lived in the South-West of Germany and were participants of a larger longitudinal study. Due to the design of the longitudinal study, the measures that are relevant for the present research were assessed at two different waves of data collection (winter 2009–2010 and summer 2011). Children's mean age was 5.11 years (*SD* = 0.62) at Wave 1 and 6.63 years (*SD* = 0.62) at Wave 2. Boys were significantly older than girls, Wave 1 *t*_(116)_ = 3.37, *p* = 0.001; Wave 2 *t*_(105)_ = 3.38, *p* = 0.001. The majority of participants (84% of mothers and 91% of fathers) described themselves as middle-class or upper middle-class. More than half of mothers (54%) and fathers (64%) had at least a college degree (in German “Fachhochschulabschluss”). The majority of parents reported having been born in Germany (86% of mothers and 88% of fathers). Accordingly, most mothers (84%) and fathers (91%) were native German speakers. Other national origins were Russia (four mothers), Romania (two mothers), Kazakhstan (two fathers) and 17 other countries represented by one participating parent each.

### Procedure

Parents were contacted via their children's preschools. They received a letter describing the aims and procedure of the study and gave written consent to participate. Confidentiality of answers was guaranteed.

At Wave 1, parental reappraisal and suppression modeling, parental supportive and non-supportive reactions and parental emotion regulation self-efficacy were assessed and information on families' socio-demographic background was collected. Parents reported on children's reappraisal and response suppression at Wave 2. Parents completed the questionnaires mostly at home, and sent them back in a prepaid envelope. Besides the measures reported in the present study, questionnaires at both waves included other self-report measures and parent measures of child temperament and behavior. At each wave of data collection, parents received a EUR 5 gift card from a local book store as a compensation for their time.

### Measures

#### Parents' reappraisal and response suppression modeling

To assess parents' reappraisal and response suppression modeling, parents reported their own use of reappraisal and response suppression. Both cognitive emotion regulation strategies were measured with the Emotion Regulation Questionnaire (ERQ; Gross and John, 2003; German version by Abler and Kessler, 2009). The ERQ contains six items to measure reappraisal (e.g., “I control my emotions by changing the way I think about the situation I am in”) and four items to measure response suppression (e.g., “I keep my emotions to myself”). Participants rated their agreement on a 7-point Likert scale from 1 (*strongly disagree*) to 7 (*strongly agree*). In the present study, confirmatory factor analyses were run to test the adequacy of a model with two uncorrelated factors for reappraisal and suppression as published by Gross and John (2003; see Abler and Kessler, 2009, for the German version of the ERQ). Modification indices suggested a covariance between the errors of items 1 and 3, two similarly worded items designed to measure reappraisal. After modeling this covariance, the model reached a satisfying fit CFI = 0.95, RMSEA = 0.06, 90% Confidence Interval (CI) [0.04, 0.09], SRMR = 0.08. Internal consistencies (see Table 1) are comparable with data on the German version of the ERQ (α = 0.76 for reappraisal and α = 0.74 for response suppression, see Abler and Kessler, 2009).

**Table 1:** **Internal consistencies of all scales used in the study**.

	**Cronbach's alpha**		
	**Total sample**	**Mothers**	**Fathers**
**PARENT VARIABLES**			
Reappraisal modeling	0.84	0.80	0.85
Response suppression modeling	0.68	0.60	0.70
Supportive reactions	0.87	0.83	0.89
Problem-focused reactions	0.72	0.66	0.76
Emotion- focused reactions	0.81	0.78	0.83
Nonsupportive reactions	0.86	0.84	0.88
Minimization	0.81	0.79	0.83
Punitive reactions	0.70	0.67	0.74
Emotion regulation self-efficacy	0.71	0.70	0.71
**CHILD EMOTION REGULATION**			
Reappraisal	0.85	0.84	0.86
Response suppression	0.71	0.70	0.71

#### Parents' supportive and non-supportive reactions

Parents reported on their reactions to the child's negative emotions by completing the Coping with Children's Negative Emotions Scale (CCNES; Fabes et al., 2002). The CCNES was translated from English to German by the last author, who is a native German speaker, and back-translated by a native English speaker trained in psychology. In the questionnaire, 12 hypothetical situations describe the child experiencing negative emotions. Each situation is followed by questions representing possible ways for parents to cope with the child's emotion, referring to different coping strategies. Parents were asked to indicate for each possible reaction how likely it would be for them to react in the way described, using a seven-point Likert scale ranging from 1 *(very unlikely)* to 7 *(very likely).*

Following McElwain et al. (2007), four subscales were used to operationalize parents' supportive reactions (*problem-focused reactions* and *emotion-focused reactions*) and non-supportive reactions (*minimization reactions* and *punitive reactions*). Parents' supportive reactions included behaviors such as if the child was nervous about “spending time at a friend's house” without the parent, the parent would “help my child think of things that he/she could do so that being at the friend's house without me wasn't scary” (problem-focused reactions) or “distract my child by talking about all the fun he/she will have with his/her friend” (emotion-focused reaction). Parents' non-supportive reactions included behaviors such as the parent would “tell my child that he/she is over-reacting and being a baby” (minimization reactions) or “tell my child that if he/she doesn't stop that he/she won't be allowed to go out any more” (punitive reactions). In the present sample, internal consistencies for the used subscales (see Table 1) were consistent with those reported in other studies with diverse samples (e.g., Fabes et al., 2002; Davidov and Grusec, 2006; Suchodoletz et al., 2011). Bivariate correlations between the problem-focused reactions and emotion-focused reactions were *r* = 0.70 (*p* < 0.001) and bivariate correlations between the minimization reactions and punitive reactions were *r* = 0.67 (*p* < 0.001). These coefficients are slightly higher than those reported by McElwain et al. (2007) for mothers and fathers separately, except for the bivariate correlation between minimization reactions and punitive reactions in fathers, which amounted to *r* = 0.80 (*p* < 0.001) in the study by McElwain et al. (2007). The supportive reactions score was computed by averaging the problem-focused and the emotion-focused reactions score. The non-supportive reactions score was computed by averaging the minimization reactions score and the punitive reactions score.

#### Emotion regulation self-efficacy

Parents' self-efficacy concerning the regulation of negative emotions were assessed at Wave 1 using the *self-efficacy in managing negative emotions* subscale of the German version of the Emotion Regulation Self-Efficacy Scale, revised version (RESE-R; Caprara et al., 2008; Gunzenhauser et al., 2013). The German version of the RESE has been shown to be reliable and valid with data from the parent sample that participated in the present study (Gunzenhauser et al., 2013). Parents rated six items concerning their beliefs about being able to regulate negative emotions (e.g., “How well can you keep from getting discouraged by strong criticism?”) on a five-point Likert scale ranging from 1 (*not at all well*) to 5 (*very well*). Cronbach's alpha for this scale (see Table 1) compares with the coefficient reported by Caprara et al. (2008) for a U.S. sample (see also Gunzenhauser et al., 2013).

#### Children's reappraisal and response suppression use

To assess children's use of reappraisal and response suppression, we adapted the German version of the ERQ (Abler and Kessler, 2009) for use as a caregiver rating (C-ERQ) and investigated its psychometric properties and validity (Gunzenhauser et al., 2012). In line with the ERQ, the C-ERQ contains six items targeting reappraisal (e.g., “It seems that my child controls his/her emotions by changing the way he/she thinks about the situation he/she is in”) and four items measuring response suppression (e.g., “It seems that my child keeps his/her emotions to himself/herself”). Parents rated their agreement on a seven-point Likert scale ranging from 1 (*strongly disagree*) to 7 (*strongly agree*). In the present study, confirmatory factor analyses revealed a good fit for the model that corresponds to the model confirmed for the adult ERQ (Gross and John, 2003; Abler and Kessler, 2009), with two uncorrelated factors for reappraisal and response suppression, CFI = 0.96, RMSEA = 0.05, 90% Confidence Interval (CI) [0.00, 0.08], SRMR = 0.06. Internal consistencies (see Table 1) compare with internal consistencies reported for the German self-rating version of the ERQ (Abler and Kessler, 2009). Mothers' and fathers' ratings of the same child's emotion regulation strategies were significantly correlated for both reappraisal, *r*_(69)_ = 0.58, *p* < 0.001, and response suppression, *r*_(70)_ = 0.38, *p* = 0.001. Paired *t*-tests showed that mothers and fathers also did not significantly differ in their absolute ratings of their child's use of reappraisal, *t*_(68)_ = −0.26, *p* = 0.794, or response suppression *t*_(69)_ = −0.31, *p* = 0.755.

### Analytic strategy

All hypotheses were investigated with regression and path analyses using Mplus version 6.12 (Muthén and Muthén, 1998–2010). In order to take into account the dependency between mother and father reports from the same family, we treated the data as clustered with mothers and fathers nested in families (Kenny et al., 2006). Dichotomous variables were dummy coded. Maximum likelihood estimation with robust standard errors (MLR) was used because MLR standard errors are robust to non-independence of observations (Muthén and Muthén, 1998–2010). The indirect effects of parents' self-efficacy on children's reappraisal and response suppression were tested using the delta method described in MacKinnon (2008), which is available in Mplus 6.12. For models with continuous observed mediation variables, such as the models tested in our study, the delta method is equivalent to the Sobel test (MacKinnon, 2008). The moderating effect of parent sex was tested using a two-group analysis approach.

#### Missing data

There was some missing data in this study, primarily due to participant dropout between the two waves of data collection (see Table 2). Analyses of patterns of missingness revealed no evidence that the pattern of missingness was dependent on any of the characteristics assessed. Missing data was handled using full information maximum likelihood.

**Table 2:** **Descriptive statistics and percentage of missing data**.

		***Mean***	***SD***	**% missing**
**PARENT VARIABLES**				
Reappraisal modeling^a^	Total (*n* = 210)	4.47	1.21	4.11
	Mothers (*n* = 111)	4.76	1.06	5.13
	Fathers (*n* = 99)	4.15	1.29	3.03
Response suppression modeling^a^	Total (*n* = 210)	3.11	1.18	4.11
	Mothers (*n* = 111)	2.75	1.02	5.13
	Fathers (*n* = 99)	3.51	1.23	3.03
Supportive reactions	Total (*n* = 211)	5.79	0.59	3.65
	Mothers (*n* = 113)	5.87	0.52	3.42
	Fathers (*n* = 98)	5.70	0.65	3.92
Nonsupportive reactions	Total (*n* = 211)	2.24	0.65	3.65
	Mothers (*n* = 113)	2.19	0.58	3.42
	Fathers (*n* = 98)	2.30	0.72	3.92
Emotion regulation self-efficacy^a^	Total (*n* = 211)	3.26	0.59	3.65
	Mothers (*n* = 113)	3.16	0.57	3.42
	Fathers (*n* = 98)	3.38	0.60	3.92
**CHILD EMOTION REGULATION**				
Reappraisal	Total (*n* = 163)	4.31	0.96	25.57
	Mothers (*n* = 93)	4.31	0.94	20.5
	Fathers (*n* = 70)	4.31	0.99	31.37
Response suppression	Total (*n* = 163)	2.16	0.97	25.11
	Mothers (*n* = 93)	2.10	0.01	20.51
	Fathers (*n* = 71)	2.23	0.91	30.39

a Variables with significant mean differences between mothers and fathers.

## Results

### Descriptive analyses

In a first step, we tested for parent sex differences in all study variables (see Table 2 for mean values). As compared to fathers, mothers reported more supportive reactions, *t*_(184.30)_ = 2.22, *p* = 0.028, more reappraisal modeling, *t*_(208)_ = 3.77, *p* < 0.001, less response suppression modeling, *t*_(190.61)_ = −4.88, *p* < 0.001, and lower emotion-regulation self-efficacy, *t*_(209)_ = −2.80, *p* = 0.006. There were no significant parent sex differences in reports on non-supportive reactions, on child reappraisal, and on child response suppression, respectively. Effects of child sex in child reappraisal (boys: *M* = 4.19, *SD* = 0.93, girls: *M* = 4.43, *SD* = 0.91) and child response suppression (boys: *M* = 2.18, *SD* = 0.87, girls: *M* = 2.14, *SD* = 1.05) were investigated using linear regressions with child sex as a predictor and family as a cluster variable in order to control for dual ratings of the same children by both mothers and fathers. Results revealed no significant differences between boys and girls. Bivariate correlations of all study measures are reported in Table 3.

**Table 3:** **Bivariate correlations of all study variables**.

	**1**	**2**	**3**	**4**	**5**	**6**	**7**
**PARENT VARIABLES**							
1. Reappraisal modeling	–						
2. Response suppression modeling	−0.02	–					
3. Supportive reactions	0.47^***^	−0.02	–				
4. Nonsupportive reactions	−0.11	0.26^***^	−0.16^*^	–			
5. Emotion regulation self-efficacy	0.24^***^	−0.14^*^	0.21^***^	0.10	–		
**CHILD EMOTION REGULATION**							
6. Reappraisal	0.34^***^	−0.08	0.34^***^	−0.16^+^	0.28^***^	–	
7. Response suppression	−0.15^+^	0.24^***^	−0.06	0.33^***^	−0.06	−0.09	–

+ p < 0.10

* p < 0.05

*** p < 0.001.

### Direct relations between emotion socialization components and children's use of emotion regulation strategies

Direct associations between emotion socialization components and children's use of reappraisal and response suppression were investigated simultaneously using multivariate multiple regression analysis. Parental supportive reactions, parental non-supportive reactions, parental reappraisal modeling and parental response suppression modeling were regressed on child reappraisal as well as on child response suppression, respectively. Parent and child sex and child age at Wave 1 were included as control variables. In order to take into account mean parent sex differences in emotion socialization components as well as correlations between diverse emotion socialization components (see Table 3), covariances between parent sex and emotion socialization components as well as covariances among emotion socialization components were included in the model. Moreover, due to the age differences between participating boys and girls reported above, child age was allowed to covary with child sex. All remaining covariances between observed independent variables were constrained to zero.

The model showed a good fit to the data, Satorra-Bentler-χ^2^_(10)_ = 9.17, *p* = 0.516, CFI = 1.00, RMSEA = 0.00, 90% CI [0.00, 0.07], SRMR = 0.03. Direct effects are reported in Table 4. For effect size measures, we computed the amounts of variance in the dependent variables explained by the model (*R*^2^). The model explained 22% of the variance in child reappraisal (*p* = 0.008) and 16% of the variance in child response suppression (*p* = 0.008).

**Table 4:** **Direct relations of emotion socialization components with children's emotion regulation strategies**.

	**Dependent variable**	
	**Child reappraisalβ**	**Child response suppressionβ**
**SOCIALIZATION COMPONENTS**		
Reappraisal modeling	0.26^**^	0.00
Response suppression modeling	−0.16^+^	0.30^***^
Supportive reactions	0.25^**^	−0.13
Nonsupportive reactions	−0.02	0.16^*^
**CONTROL VARIABLES**		
Parent sex^a^	0.14^*^	−0.03
Child sex^a^	−0.16^+^	−0.02
Child age	−0.03	0.02

a 0 = female and 1 = male. The following standardized covariances were significant: cov (reappraisal modeling, supportive reactions) = 0.47, p < 0.001, cov (response suppression modeling, non-supportive reactions) = 0.26, p < 0.001, cov (supportive reactions, non-supportive reactions) = −0.16, p = 0.036.

+ p < 0.10

* p < 0.05

** p < 0.01

*** p < 0.001.

### Indirect relations between parents' emotion regulation self-efficacy and children's use of emotion regulation strategies

**Figure 2:**
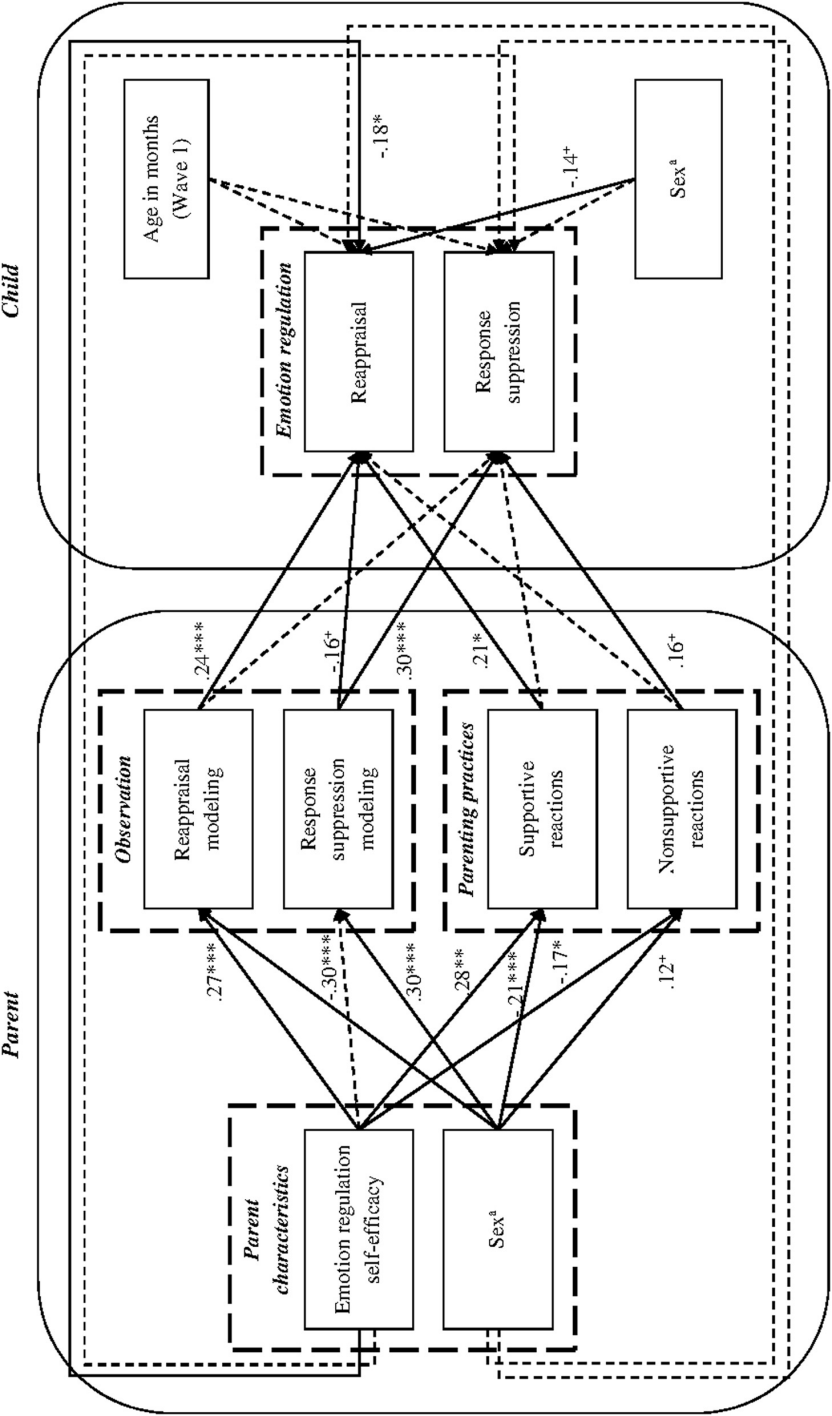
**Direct and indirect relations between parents' emotion-regulation self-efficacy, socialization components, and children's use of emotion regulation strategies**. ^a^0 = female and 1 = male. Solid lines represent significant effects; dashed lines represent non-significant effects. Covariances are not displayed in the figure to increase clarity. The following standardized covariances were significant: *cov* (reappraisal modeling, supportive reactions) = 0.40, *p* = 0.001; *cov* (response suppression modeling, non-supportive reactions) = 0.26, *p* = 0.001. ^+^*p* < 0.10, ^*^p < 0.05, ^**^p < 0.01, ^***^p < 0.001.

Indirect associations between parents' emotion regulation self-efficacy and children's reappraisal and response suppression use were investigated using path analysis (see Figure 2). In a path analysis, a variable (in our case, emotion socialization components) can be modeled as a dependent variable in one relationship and as an independent variable in another (Muthén and Muthén, 1998–2010). All emotion socialization components were regressed on parent emotion regulation self-efficacy while controlling for parent sex. Child reappraisal and response suppression were regressed on all emotion socialization components, controlling for parent and child sex and child age at Wave 1. Moreover, direct relations between parent emotion regulation self-efficacy and later child reappraisal and response suppression were included in the model to be able to assess whether emotion socialization components fully mediate the relationship between emotion regulation self-efficacy and child emotion regulation strategies. All covariances between independent variables were set to zero, with the exceptions of the covariances among emotion socialization components and the covariance between child sex and age. The model showed a good fit to the data, Satorra-Bentler-χ^2^_(12)_ = 9.04, *p* = 0.700; CFI = 1.00, RMSEA = 0.00, 90% CI [0.00, 0.05], SRMR = 0.03). In line with our expectations, the delta method revealed a significant positive indirect effect of parents' emotion regulation self-efficacy on children's reappraisal, β = 0.12, *SE* = 0.05, *p* = 0.014. This effect was mainly due to a significant specific indirect effect through parents' reappraisal modeling (β = 0.06, *SE* = 0.03, *p* = 0.027), while there was also a trend pointing to a specific indirect effect through parents' supportive reactions (β = 0.06, *SE* = 0.03, *p* = 0.076). There were no significant specific indirect effects of parental emotion regulation self-efficacy on child reappraisal through parental non-supportive reactions or parental response suppression modeling. The total and specific indirect effects of parents' emotion regulation self-efficacy on children's response suppression were not significant. The variance in children's reappraisal explained by the model was 25% (*p* = 0.003), and the variance explained in children's response suppression was 16% (*p* = 0.009).

### Parent sex as a possible moderator

Possible moderating effects of parent sex in the paths between parent emotion regulation self-efficacy, emotion socialization components, and child reappraisal and response suppression were tested using multiple group analyses (see Vandell et al., 2010; Otterpohl et al., 2012). Mothers and fathers were treated as two subgroups. Building on the model shown in Figure 2 (but omitting parent sex as a control variable), two models were estimated. The unconstrained model allowed for parent sex-specific path coefficients between parental emotion regulation self-efficacy, emotion socialization components, and child reappraisal and response suppression. In contrast, the constraint model restricted these paths to be equal for mothers and fathers. A Satorra-Bentler scaled chi-square difference test comparing both models was not significant, Δχ^2^_(18)_ = 16.91, *p* = 0.529 (see Satorra, 2000). This indicates that the model assuming equal path coefficients for mothers and fathers is preferable. Thus, parent sex did not moderate any direct or indirect effects of parent emotion regulation self-efficacy or parent emotion socialization components.

## Discussion

This study investigated parental socialization of two cognitive emotion regulation strategies, namely reappraisal and response suppression within the framework of the tripartite model of emotion socialization with a German sample. Both maternal and paternal emotion socialization were investigated. Focusing on parental emotion-related beliefs and behaviors, direct and indirect relations with child reappraisal and response suppression use were examined. Additionally, the moderating role of parent sex was explored.

### Direct relations between emotion socialization components and children's use of emotion regulation strategies

Consistent with previous theorizing on modeling as a socialization component (Bandura, 1977), findings of the present study suggest that parents' modeling of reappraisal might facilitate reappraisal in their children, and parents' modeling of response suppression use might facilitate response suppression in their children. These findings extend previous results (Bariola et al., 2011) demonstrating that mothers' own response suppression use relates to their adolescents' response suppression use but no such effect was found for reappraisal use. While Bariola et al. (2011) argue that for adolescents parental response suppression might be more readily observable than parental reappraisal, this might not hold true for children in early and middle childhood. During childhood, parent-child conversations about emotions, including explicit verbalization of emotion regulation strategies, may help children notice reappraisal use in their parents (Thompson, 1991; Thompson and Meyer, 2007). Notably, the present study also revealed a tendency for parental response suppression modeling to be directly related to lower later reappraisal use in their children. This result adds to the evidence that parental response suppression might hinder children's acquisition of emotion regulation strategies that require different skills than response suppression, such as reappraisal (see Silk et al., 2006). In order to be able to reappraise a situation, a child needs to focus on the emotional experience. This might be impeded when his or her attention is drawn to showing the adequate emotional display by a parents' frequent and strong response suppression modeling. In contrast, parental reappraisal modeling was not related to child response suppression. Future research should specify whether there is a specific association between parental response suppression modeling and lower child reappraisal, or whether this finding generalizes to parental modeling of overall maladaptive emotion regulation strategies hindering children's use of overall adaptive strategies. From a methodological perspective, future research needs to consider a possible dependency between parents' preferred emotion regulation strategies and the quality of their reports on their own and their children's emotion regulation strategies. For instance, research by Gottman et al. (1996) suggests that parents who use response suppression frequently might be less insightful observers of their children's emotions and regulatory attempts than parents who seldom use response suppression. For the present research, this implies a possible bias in findings concerning relations between parental suppression and child emotion regulation strategies. However, the simultaneous analysis of all data in a path model allowed for statistical control of parental suppression in all other paths. Therefore, estimates of the relations of all other emotion socialization components to child emotion regulation strategies are unlikely to be biased by individual differences in parents' suppression.

Based on previous research investigating parental reactions to children's emotions and their relationship to child emotion regulation, it seemed plausible that child reappraisal might be promoted by both parental supportive reactions and a lack of parental non-supportive reactions, and that child response suppression might be enhanced by both parental non-supportive reactions and a lack of supportive reactions (Eisenberg et al., 1998). However, results of the present study revealed that supportive parental reactions (and not a lack of non-supportive reactions) facilitate child reappraisal, and non-supportive parental reactions (and not a lack of parental supportive reactions) facilitate child response suppression. These results extend findings by Jaffe et al. (2010), who found higher perceived parental care to be related to both higher reappraisal and lower response suppression use in adolescent children. As described above, Jaffe et al. (2010) treated parental care as a unidimensional construct and were therefore unable to distinguish influences of supportive and non-supportive parenting practices. The present study adds to the evidence for two separate (though negatively related) dimensions of supportive and non-supportive reactions with distinct psychological functions in emotion socialization (Eisenberg et al., 1998; Fabes et al., 2002; Davidov and Grusec, 2006).

### Indirect relations between parents' emotion regulation self-efficacy and children's use of emotion regulation strategies

In support of the tripartite model, our results revealed an indirect effect of parent emotion regulation self-efficacy on children's later reappraisal use through parents' reappraisal modeling and supportive reactions. However, there was also a significant direct effect of parents' emotion regulation self-efficacy on child reappraisal, indicating partial mediation. This might be due to mediating influences of socialization components not modeled in the present study, for instance, family climate (Morris et al., 2007). No significant indirect effect of parents' emotion regulation self-efficacy on child response suppression was detected in the present study. This result might be due to two counteracting tendencies in the relationship between emotion regulation self-efficacy, non-supportive reactions, and child response suppression (see Figure 2): while higher parental emotion regulation self-efficacy is negatively related to non-supportive reactions, non-supportive reactions show a tendency of being positively related to child response suppression. It might be possible that the two opposite influences result in a failure to detect any significant effect of emotion regulation self-efficacy (MacKinnon, 2008). Future research should investigate this possibility. Taking together our analyses on both direct and indirect relations, the majority of significant results were found within the domains of either overall favorable processes (i.e., effects of parental reappraisal modeling, supportive reactions, and emotion regulation self-efficacy on child reappraisal) or overall unfavorable processes (i.e., effects of response suppression modeling and non-supportive reactions on child response suppression). This pattern of results corresponds with findings by Otterpohl et al. (2012), who found indirect effects of parental strain (i.e., an overall unfavorable factor) on children's maladaptive emotion regulation strategies, but not on children's adaptive strategies. Thus, parental influences on children's cognitive emotion regulation strategies might be domain-specific: one the one hand, parental positive resources might facilitate overall adaptive cognitive emotion regulation strategies through modeling of overall adaptive emotion regulation strategies and supportive reactions. On the other hand, parental burdens might facilitate overall maladaptive cognitive emotion regulation strategies through modeling of overall maladaptive emotion regulation strategies and non-supportive reactions. Future research should investigate these relations for families from non-Western cultures, for whom the socialization components and emotion regulation strategies investigated in the present study might carry different valences due to culture-specific values and expectations (Butler et al., 2007).

### Parent sex differences

A third aim of the present study was the exploration of moderating effects of parent sex. As no significant moderation effects occurred, it can be concluded that, at least in our data, these pathways are equal for mothers and fathers. These results contrast with previous theorizing which suggested different processes for mothers and fathers due to greater involvement of mothers in their children's emotion socialization or due to less scripted behavioral schemes for fathers as compared to mothers (McDowell et al., 2002; Wong et al., 2009). However, findings align with recent empirical work showing no general or consistent pattern of parent sex differences across diverse aspects of emotion socialization (McElwain et al., 2007; Wong et al., 2009; Baker et al., 2011; Liang et al., 2012). Possibly, the non-existence of parent sex differences in pathways of emotion socialization found in the current study may reflect changes in the ways mothers and fathers define their roles in the family. As fathers may have become more involved in the parenting of young children, role expectations for mothers and fathers may converge in some respect, emphasizing sensitive caregiving as a desirable behavior for both parents (see Petteri Eerola and Huttunen, 2011). Given that previous studies found parent sex differences for the socialization of specific emotion-related constructs but not for others (McElwain et al., 2007; Wong et al., 2009), findings of the present study might be specific to the socialization of reappraisal and response suppression, and they might not necessarily generalize to other emotion regulation strategies. However, further research is needed to clarify parent sex differences in various domains of socialization. Also, the present study focused on the regulation of negative emotions in general. It seems possible that parent sex differences might exist with regard to reappraisal or suppression of specific emotions within the broader area of negative emotions (e.g., anger vs. fear). This might be relevant to specific adjustment difficulties such as externalizing and internalizing symptoms (Chaplin et al., 2005). In order to reach more conclusive insight on parent sex differences in emotion socialization in general, meta-analytic work seems necessary. Additionally, a next step might include a more in-depth focus on the role of sex in parental socialization of reappraisal and response suppression. While taking child sex into account, potential differences between same-sex parent-child dyads vs. opposite-sex parent-child dyads for both mothers and fathers could be explored (Chaplin et al., 2005; McElwain et al., 2007).

### Limitations

The present study has several limitations to be considered when interpreting the results. First, we solely relied on questionnaire measures, which might be sensitive to social desirability and response tendencies. Moreover, the current study relied on a single source, that is, parent self-reports and parent reports on their children's emotion regulation strategies. It seems thus likely that relations between parent variables and child variables might be overestimated in our data due to common source variance. This might apply in particular to similarities between parents' ratings of their own emotion regulation strategies and their ratings of their children's emotion regulation strategies because both measures were parallel in wording. In our study, possible biases due to the common source variance might however have been reduced by the fact that there was an interval of one and a half year between parents' self-rating and parents' rating of their children, which prevented parents from directly comparing their reports on themselves and their reports on their children. Moreover, the correspondence between mothers' and fathers' ratings of the same child's emotion regulation strategies (as indicated by both non-significant mean differences tests and significant correlations) provides further support for the validity of parents' ratings of their child. To our knowledge, there are so far no alternative valid measures to assess parents' and children's habitual reappraisal and response suppression use or parental emotion regulation self-efficacy (see Bariola et al., 2012). In particular, efforts to create self-rating measures to assess the frequency of specific cognitive emotion regulation strategies in childhood have not yet been successful, suffering from low reliability and a lack of relations to other measures (see Otterpohl et al., 2012). An alternative approach for the present study might have involved other-ratings by other adults than the respective parent. Specifically, a promising and feasible approach for future studies might consist in using one parent's self-reported emotion-related beliefs and behaviors as a predictor for child outcomes as rated by the other parent. On the other hand, on the basis of general behavioral tendencies, children may also modify their cognitive emotion regulation strategies depending on the interaction partners present (Zeman et al., 1997). Insofar, reactions to parental socialization might be particularly prominent in the presence of this very parent. Therefore, this parent would have the best opportunities to observe influences of his/her behavior on the child. Nevertheless, future research should make an effort to develop and validate measures to assess the use of cognitive emotion regulation strategies in childhood in order to strengthen study designs by including multiple sources. In any case, a follow-up study to the present research might attempt to replicate the present results while assessing emotion-related parenting practices with an observational tool (e.g., Spinrad et al., 2004; Cassano and Zeman, 2010). Moreover, a valuable addition would consist in including child reports on parental behavior. Although empirical comparisons between child reports and parent self-reports on parenting are scarce for preschool children, research with elementary students and adolescents shows low to moderate correlations between parent and child reports of parenting (Gaylord et al., 2003; Barry et al., 2008). Findings on adolescents' ratings of parenting and their self-reported emotion regulation strategies indicate that children's perceptions of parenting might be predictive of their reappraisal and response suppression use (Jaffe et al., 2010).

Another limitation of the current study lies in the interval of one and a half year between the assessment of parent variables and the measurement of child emotion regulation strategies. As mentioned above, this time lag might have contributed to reducing a potential overestimation of the similarities between parents and their children. However, this design did not allow us to control for other factors that might have influenced children's development of emotion regulation strategy use in the meantime, for instance, changes due to maturation. Our analyses are correlational in nature and thus do not take into account the absolute frequency of children's reappraisal or response suppression use but exploit individual differences, that is, the frequency of reappraisal and response suppression relative to other children. Thus, maturational influences would pose a problem to our analyses especially if they implied instability of individual differences in emotion regulation strategy use over time. To our knowledge, there is so far no empirical investigation of the stability over time of individual differences in reappraisal and response suppression use in childhood. However, other aspects of emotional competence and emotion regulation have been shown rather stable over the preschool period (Eisenberg, 2000; Halligan et al., 2013). It thus seems plausible that this might hold true for reappraisal and response suppression use as well. Although caution is in order, this might justify the assumption that the time lag in our study might not have biased our results severely.

Reappraisal and response suppression were measured as comprehensive strategies for the regulation of positive and negative emotions. This is in line with studies showing that individual differences in the habitual use of reappraisal and response suppression are stable across positive and negative emotions (Gross and John, 2003). In contrast, parental reactions to children's emotions and parental emotion regulation self-efficacy were measured with a focus on negative emotions. The emphasis of negative emotions corresponds with the finding that most instances of everyday emotion regulation target negative emotions (Gross et al., 2006). However, regulation of positive emotions has been related to adjustment in two aspects: on the one hand, down-regulation of exuberant positive emotions has been associated with less behavior problems and more prosocial behavior in children; on the other hand, up-regulation of positive emotions has been suggested to promote resilience to stressful events (Rydell et al., 2003; Tugade and Fredrickson, 2007). Thus, future research should additionally take the regulation of positive emotions into account.

In terms of statistical analyses, our study used path models, which do not consider measurement errors. This problem could be counteracted by using structural equation modeling (SEM). However, given the relatively high item number of the questionnaires used (e.g., the CCNES contains 24 items respectively for supportive and non-supportive reactions), our sample was not large enough to allow for reliable SEM estimation (Tabachnick and Fidell, 2007). Therefore, the effect sizes found in our study have to be interpreted with caution. Also, indirect effects were tested with the Sobel test without applying the bootstrapping procedure preferred by many researchers (see Preacher and Hayes, 2004). Bootstrapping is not available in Mplus 6.12 in combination with a clustering variable (for parents nested in families). However, results of simulation studies indicate that MLR parameter estimates and standard errors as used in the present study are identical to those obtained by bootstrapping (Muthén and Muthén, 1998–2010, p. 548). Moreover, the rate of missing data was relatively high, especially considering child emotion regulation strategies. Although there were no indications of systematic dropout and despite the application of full information maximum likelihood in order to fully use the available information, this limitation should be kept in mind when interpreting our results.

Finally, only selected aspects of the tripartite model were examined in the present study. Modeling, parenting practices, and emotion regulation self-efficacy were chosen for investigation as they represent John and Gross' (2004) emphasis of individual parents' emotion-related beliefs and behaviors in the socialization of reappraisal and response suppression. However, future research should examine whether the consideration of family climate, further parent characteristics, and bidirectional influences add to the understanding of children's development of reappraisal and response suppression use.

### Conclusions and implications

Taken together, this study confirmed that the tripartite model of emotion socialization (Morris et al., 2007) is applicable to the socialization of the cognitive emotion regulation strategies of reappraisal and response suppression, and adds to the evidence for John and Gross' (2004) suggestions about the role of parental emotion-related beliefs and behaviors in this process. Findings did not reveal any differences between maternal and paternal socialization paths. Specifically, our results showed that it is not merely the behavioral level (i.e., emotion socialization components) that matters in emotion socialization, but that these behaviors need to be understood within a wider framework of parent characteristics (Gottman et al., 1996; John and Gross, 2004; Morris et al., 2007). Although our findings are so far preliminary and did not focus on interventions, practical implications seem evident: interventions which aim to help parents in the socialization of overall adaptive cognitive emotion regulation strategies should include more than support on the behavioral level; they should also help parents reflect their beliefs on emotion regulation. Remarkably, our results indicated that parental influences on children's cognitive emotion regulation strategies might function within the domains of either overall adaptive or overall maladaptive processes. If future research confirms this suggestion, it seems necessary for parent-child interventions to focus on both the promotion of overall adaptive cognitive emotion regulation strategies and the prevention of overall maladaptive cognitive emotion regulation strategies. In our introductory example, this might imply that it would not be sufficient to just convince Jacob's mother that her son should be allowed to show his feelings. Instead, Jacob's mother should be encouraged to coach her son in more adaptive ways of facing and regulating emotions, such as using reappraisal where it is appropriate. Over and above the specific cognitive emotion regulation strategies investigated in this study, such an approach might contribute to a socialization of overall adaptive emotion regulation strategies that promote children's long-term health and life satisfaction.

### Conflict of interest statement

The authors declare that the research was conducted in the absence of any commercial or financial relationships that could be construed as a potential conflict of interest.
